# Potential of ASCOT-Carer for evaluating the quality of life of family caregivers for patients with Alzheimer’s disease in Japan

**DOI:** 10.1007/s11136-026-04252-6

**Published:** 2026-05-03

**Authors:** Shinichi Noto, Kentaro Yamato, Keisuke Onuki, Tomohiro Kondo

**Affiliations:** 1 Department of Rehabilitation, Niigata University of Health and Welfare, Niigata, Japan; 2 Medical Affairs, Otsuka Pharmaceutical Co., Ltd, Tokyo, Japan; 3 Department of Public Health, Graduate School of Medicine, Juntendo University, Tokyo, Japan

**Keywords:** Alzheimer’s disease, ASCOT-Carer, Caregivers, EQ-5D-5L, Japan, Quality of life

## Abstract

**Purpose:**

We explored the suitability of the Adult Social Care Outcomes Toolkit for Carers (ASCOT-Carer) against the widely used EQ-5D for evaluating the quality of life (QoL) of Japanese caregivers of patients with Alzheimer’s disease. This research aimed to expand the available preference-based measures for assessing caregiver QoL in health economic evaluations in Japan.

**Methods:**

This was a cross-sectional survey using a web-based questionnaire. The alignment between the ASCOT-Carer and established measures, as well as its construct alignment, were evaluated. These were respectively investigated through Pearson’s correlation analysis with the EQ-5D-5L and factor analysis using the EQ-5D-5L and the eight-item short Japanese version of the Zarit Burden Interview (J_ZBI-8).

**Results:**

In total, 705 live-in family caregivers of patients with Alzheimer’s disease completed the main survey. The absolute correlation coefficients were between J_ZBI-8 and EQ-5D-5L scores, + 0.268; between J_ZBI-8 and ASCOT-Carer scores, + 0.472; and between EQ-5D-5L and ASCOT-Carer scores, + 0.463. Factor analysis revealed that the only factors showing moderate/stronger correlations with factors constituted by J_ZBI-8 were those derived from ASCOT-Carer (inter-factor correlation: +0.31 and + 0.50). The ASCOT-Carer was a more important variable relative to the EQ-5D-5L for measuring caregivers’ QoL related to caregiver burden.

**Conclusion:**

The ASCOT-Carer may be a more suitable tool for evaluating the impact of caregiving burden on caregivers’ QoL. When incorporating QoL based on caregiver burden in health economic evaluations, the ASCOT-Carer may be an appropriate option.

**Supplementary Information:**

The online version contains supplementary material available at 10.1007/s11136-026-04252-6.

## Abstract (plain language summary)

Caring for a family member with Alzheimer’s disease can be physically, emotionally, and mentally challenging, affecting a caregiver’s overall well-being and daily life. We looked at two tools—the Adult Social Care Outcomes Toolkit for Carers (ASCOT-Carer) and the EQ-5D-5L—to see how well they measure the impact of caregiving on a caregiver’s quality of life and to expand the available tools for properly evaluating caregiver quality of life in health economic evaluations in Japan. We also examined how these tools related to caregivers’ feelings of stress and burden using the eight-item short Japanese version of the Zarit Burden Interview. We surveyed 705 family caregivers of people with Alzheimer’s disease through an online questionnaire. Our results showed that both tools provide helpful information but focus on different areas of a caregiver’s quality of life. The EQ-5D-5L mainly looks at general health issues like mobility, self-care, and anxiety, but it doesn’t fully capture the social and emotional challenges of caregiving. Alternatively, the ASCOT-Carer offers a clearer picture of a caregiver’s well-being, especially regarding social connections and emotional health. Moreover, the ASCOT-Carer was better than the EQ-5D-5L at showing the stress and burden that caregivers experience. This highlights that the ASCOT-Carer is more sensitive to the specific challenges that caregivers face. While both tools are helpful, the ASCOT-Carer may be a more suitable tool for evaluating the impact of caregivers’ real-life struggles on their quality of life, making it a valuable resource for health economic evaluations.

**Supplementary Information:**

The online version contains supplementary material available at 10.1007/s11136-026-04252-6.

## Introduction

In 2019, approximately 57 million individuals globally were living with dementia, a number expected to increase to 153 million by 2050 [1]. In Japan, this figure is projected to reach 7 million by 2025 [2]. Most care for patients with dementia and Alzheimer’s disease (AD) is provided by family members (spouses or adult children) and other informal caregivers [3–5]. AD, which accounts for 60% to 80% of dementia cases, considerably burdens these caregivers, increasing their risk of anxiety, depression, and sleep disorders [6–8]. Caregivers often miss work, experience low productivity, and depend heavily on medical resources [6, 9]. Overall, AD imposes considerable costs and health spillover on caregivers [9, 10].

Despite growing recognition of caregiver outcomes in AD, spillover effects, which refer to the impact of a patient’s condition and required care on family members [11–13], are rarely included in health economic evaluations. Although expert panels have recommended including caregiver spillover effects from a social perspective in health economic evaluations [14–16], implementation remains challenging because of lack of data on preference-based measures (PBMs) for spillover effects and lack of comparative data to quantify their magnitude [17]. Ongoing debate also surrounds the scope of spillover effects to include in health economic evaluations [18–20]. Finally, if caregivers are unable to distinguish their own burden or health changes from those attributable to the patient’s condition, caregiver-related effects may be inadvertently incorporated into patient-reported quality of life (QoL), which could therefore result in double counting [16, 21]. Accordingly, selecting a preference-based measure that appropriately captures caregiving-related impact on caregivers’ QoL is important.

Medical and social care interventions targeting patients may affect caregiver outcomes [22]. For example, some interventions may reduce the physical and emotional demands on caregivers, while others may improve patient outcomes but negatively impact caregiver well-being [23]. Therefore, excluding spillover effects may lead to underestimating or overvaluing medical interventions with broad societal benefits [24, 25]. However, few studies have included spillover effects in their analyses. Most analyses considering spillover effects are cost-related, and even fewer consider the combined health benefits, especially quality-adjusted life years, for caregivers [17, 24, 26].

For caregivers of patients with dementia, caregiver burden is typically assessed using the Zarit Burden Interview (ZBI) [27]. However, few assessment tools focus on health-related QoL [28]. Although the EQ-5D is commonly used to assess QoL [17], it has been suggested that the EQ-5D has limited sensitivity in measuring caregiver QoL [29]. The five dimensions measured by the EQ-5D may not adequately reflect caregiver concerns or broader aspects of QoL that may be affected by medical and social care interventions [29].

In response to these challenges, several PBMs have been developed to measure the QoL of informal caregivers. For example, the CarerQoL was developed in the Netherlands to assess the health-related QoL of informal caregivers [30]. The Care Experience Scale (CES) was also developed in the UK to reflect caregivers’ care experiences in economic evaluations [31]. However, neither the CarerQoL nor CES has been adapted to a Japanese version with value sets for Japan.

The Adult Social Care Outcomes Toolkit for Carers (ASCOT-Carer) is a PBM developed in the UK to evaluate the social care-related QOL (SCRQoL) of informal caregivers [32]. It has been translated and cross-culturally adapted for use in Japan using internationally recognized best-practice guidelines to ensure conceptual and measurement equivalence [33–36]. Psychometric evaluation using combined factor analysis and item response theory supported a single-factor structure, an appropriate response scheme, and satisfactory validity, as evidenced by item discrimination and difficulty parameters within expected ranges [37]. In addition, preference weights based on Japanese social preferences have also been developed, making it a PBM that can be used for economic evaluations in Japan [34]. A comparative study of the ASCOT-Carer and EQ-5D for unpaid informal caregivers suggested that these measures capture different concepts, indicating that the ASCOT-Carer is more useful than the EQ-5D for measuring caregiver QoL [38]. However, to date, no studies have measured caregiver QoL using the Japanese ASCOT-Carer among caregivers of patients with AD in Japan.

The purpose of this study was to compare whether the ASCOT-Carer or EQ-5D-5L more appropriately captures caregiver QoL related to caregiver burden (J_ZBI-8) among Japanese live-in family caregivers of patients with AD. This study aimed to investigate the potential of ASCOT-Carer (a patient characteristics-based measurement tool) as a feasible PBM for assessing caregivers’ QoL in health economic evaluations in Japan.

## Methods

### Study design and participants

This was a cross-sectional survey using a web-based questionnaire. The study participants were recruited from an opt-in online survey panel managed by Macromill, Inc. (Tokyo, Japan). Survey invitations were distributed to panel members who had registered as caregivers, irrespective of the reason for caregiving. Participants who provided consent to participate in the survey via an on-screen consent form were provided a screening survey, which included three questions [39]. The survey covered aspects of caregiver demographics and caregiving characteristics, caregiver burden (J_ZBI-8), SCRQoL (ASCOT-Carer), and health-related QoL (EQ-5D-5L). The screening questions were initially offered to determine which respondents met the inclusion criteria. Participants who passed the screening were then asked to complete the main survey, which included 43 questions [39]. The main survey period was from November 13 to 27, 2023.

Otsuka Pharmaceutical Co., Ltd. financially compensated all participants for participating in this study via point-based compensation credited to participants’ registered accounts (through the survey provider’s system). Regarding participant compensation, participants received points based on overall survey completion. Participants who completed only the screening questions (including those who were excluded) received 2 points, whereas participants who completed the main survey received up to a maximum of 82 points. The total number of points awarded varied depending on survey branching, as some questions were conditionally skipped based on prior responses. Compensation was not contingent on specific answers.

The study participants were live-in family caregivers of patients with AD who were registered as disease monitors with Macromill, Inc., 19–79 years of age, within the second degree of kinship, including common-law family members, and the primary or partial caregiver of the patient with AD. Participants who closed the web browser while completing the questionnaire were excluded from this study. 

The study protocol was approved by the Research Ethics Committee of Otsuka Pharmaceutical Co., Ltd. (Reception number: 230928), and the study was conducted in accordance with the Declaration of Helsinki and adhered to Ethical Guidelines for Medical and Biological Research Involving Human Subjects. All study participants provided written informed consent. This study was registered at the University hospital Medical Information Network Clinical Trials Registry (UMIN000053306).

### Measurement of caregiver burden and QoL

In this study, the following three scales were used: the eight-item short Japanese version of the ZBI (J_ZBI-8) [40] to evaluate caregiver burden; the ASCOT-Carer [32, 41] to evaluate social care-related QoL; and the EQ-5D-5L [42] to evaluate health-related QoL.

### J_ZBI-8

The J_ZBI-8 is a concise assessment scale designed to evaluate the burden of caregivers who care for patients with chronic diseases or disabilities. It is a shortened and restructured version of the original ZBI, retaining the eight main items that comprehensively capture the emotional, physical, and social burdens faced by caregivers while reducing the response burden. It focuses on core aspects such as stress, guilt, and fatigue, enabling rapid and reliable measurement in clinical research settings. Each of the eight items is rated on a 5-point Likert scale, and the total burden score is calculated as the sum of individual item scores, ranging from 0 to a maximum of 32 points. For clarity and consistency, in analysis, eight items from the original Zarit Burden Interview (ZBI), specifically Q4, Q5, Q6, Q9, Q12, Q13, Q18, and Q19, were renumbered as Z1 through Z8 for the purposes of this study [40].

### ASCOT-Carer

The ASCOT-Carer is a PBM developed in the UK in 2015 to measure the SCRQoL of informal caregivers caring for adults receiving social care. SCRQoL refers to aspects of QoL that may be affected by adult social care services. This scale consists of the following seven domains: A1, occupation; A2, control over daily life; A3, self-care; A4, personal safety; A5, social participation and involvement; A6, space and time to be yourself; and A7, feeling encouraged and supported. Each domain has response levels ranging from 1 to 4, and the preference weights for each response level were calculated in 2022 using the best–worst scaling method in a study of the general Japanese population. In this study, we used the Japanese version of the value set and calculated the index score by adding the preference weights corresponding to the response levels for each domain. This score is rescaled from 0 to 1, where 0 represents the worst state of SCRQoL and 1 represents the best state of SCRQoL [32, 41].

### EQ-5D-5L

EQ-5D-5L is a standardized PBM developed by the EuroQol Group in 2011 to assess health-related QoL, consisting of the following five domains: E1, mobility; E2, self-care; E3, usual activities; E4, pain/discomfort; and E5, anxiety/depression [42]. Each domain has five response levels, and each response level is assigned a preference weight. These preference weights are developed in each country, and in Japan, they were derived in 2016 using the time trade-off method in a study targeting the general population [43]. In this study, using the Japanese version of the value set, the index score was calculated by subtracting the sum of the preference weights corresponding to each domain’s response level and a constant term from the base score 1. This score ranges from 0 (equivalent to death) to 1 (perfect health) and may be less than 0 (health states considered worse than death) depending on the health status [42, 43].

### Statistical methods

Data obtained in a separate study were used for sample size calculations [44]. For background characteristics, categorical variables were summarized using n (%); continuous data were summarized using mean, standard deviation (SD), quartiles, maximum, and minimum values. The gender of participants was defined based on self-report.

The alignment between the ASCOT-Carer and other established measures was evaluated based on the total score and the correlation between the score of each domain and the EQ-5D-5L score. Multivariable analysis was conducted to determine the association between caregiver burden (J_ZBI-8) and QoL scales for caregivers of patients with AD (ASCOT-Carer and EQ-5D-L). The relationship between the QoL scales, ASCOT-Carer and EQ-5D-5L, was based on the index score and Pearson’s product-rate correlation coefficients between each domain. The correlation coefficient was interpreted as weak correlation (< 0.3), moderate correlation (≥ 0.3 to < 0.5), and strong correlation (≥ 0.5) according to Spearman’s correlation coefficient [45]. In addition, factor analysis was conducted to explore the empirical clustering of items across the domains of the QoL scales (ASCOT-Carer and EQ-5D-5L) and the caregiver burden (J_ZBI-8) [46, 47].

Factor analysis was conducted following an assessment of sampling adequacy using the Kaiser–Meyer–Olkin procedure. The measure of sample validity of ≥ 0.8 confirmed that factor analysis was possible. Bayesian Information Criterion (BIC) was used to determine the appropriate number of factors. Seven factors were selected based on the BIC, which was adjusted for sample size. Oblique rotation (Oblimin) was specified for estimating factor loadings, as correlations among factors were assumed, and the maximum likelihood estimation was used as the estimation method. In interpreting the factors, factor loadings ≥ 0.3 were considered to indicate a meaningful correlation [48]. These data contained no missing values, eliminating the need for substitution or additional processing.

The regression analyses were designed to compare the extent to which two caregiver QoL measures (ASCOT-Carer and EQ-5D-5L) capture the impact of caregiving burden, given prior evidence that generic health-related QoL instruments such as the EQ-5D may have limited sensitivity to caregiving effects [49]. Accordingly, regression analysis adjusted for the same covariates was performed. It was hypothesized that higher caregiver burden (J_ZBI-8) would be associated with lower QoL for both measures, and that the strength of this association would be greater for ASCOT-Carer than for EQ-5D-5L. It was also hypothesized that the ASCOT-Carer would contribute more to the explained variance in caregiver burden than the EQ-5D-5L when both measures were included (assessed using the Lindeman, Merenda, and Gold [LMG] index). A general linear regression model was used for estimating the association between caregiver burden and the QoL scales (ASCOT-Carer and EQ-5D-5L), adjusting for six variables (1, patient age; 2, patient gender; 3, caregiver age; 4, caregiver gender; 5, caregiver’s employment status [yes/no]; and 6, nursing time). Caregiver burden was used as the independent variable, and the QoL scale was used as the primary dependent variable. Point estimates and 95% confidence intervals (CIs) for the primary dependent variable were calculated. To evaluate the importance of the ASCOT-Carer and EQ-5D-5L in measuring the burden of care, both indices were incorporated into the model, and the LMG index was calculated [50]. In other words, the difference between the overall and individual determination coefficients is large, and the larger the overall determination coefficient, the larger the LMG.

The analysis results were independently double-programmed by two statisticians from Macromill, Inc., and no discrepancies were found. Furthermore, a statistician from Otsuka Pharmaceutical Co., Ltd., confirmed the results. Additional analysis by two statisticians from Otsuka Pharmaceutical Co., Ltd., reproduced the results using R 4.2.3 and JMP^®^17 (JMP Statistical Discovery LLC, Cary, NC, USA).

## Results

### Study participants

**Fig. 1:**
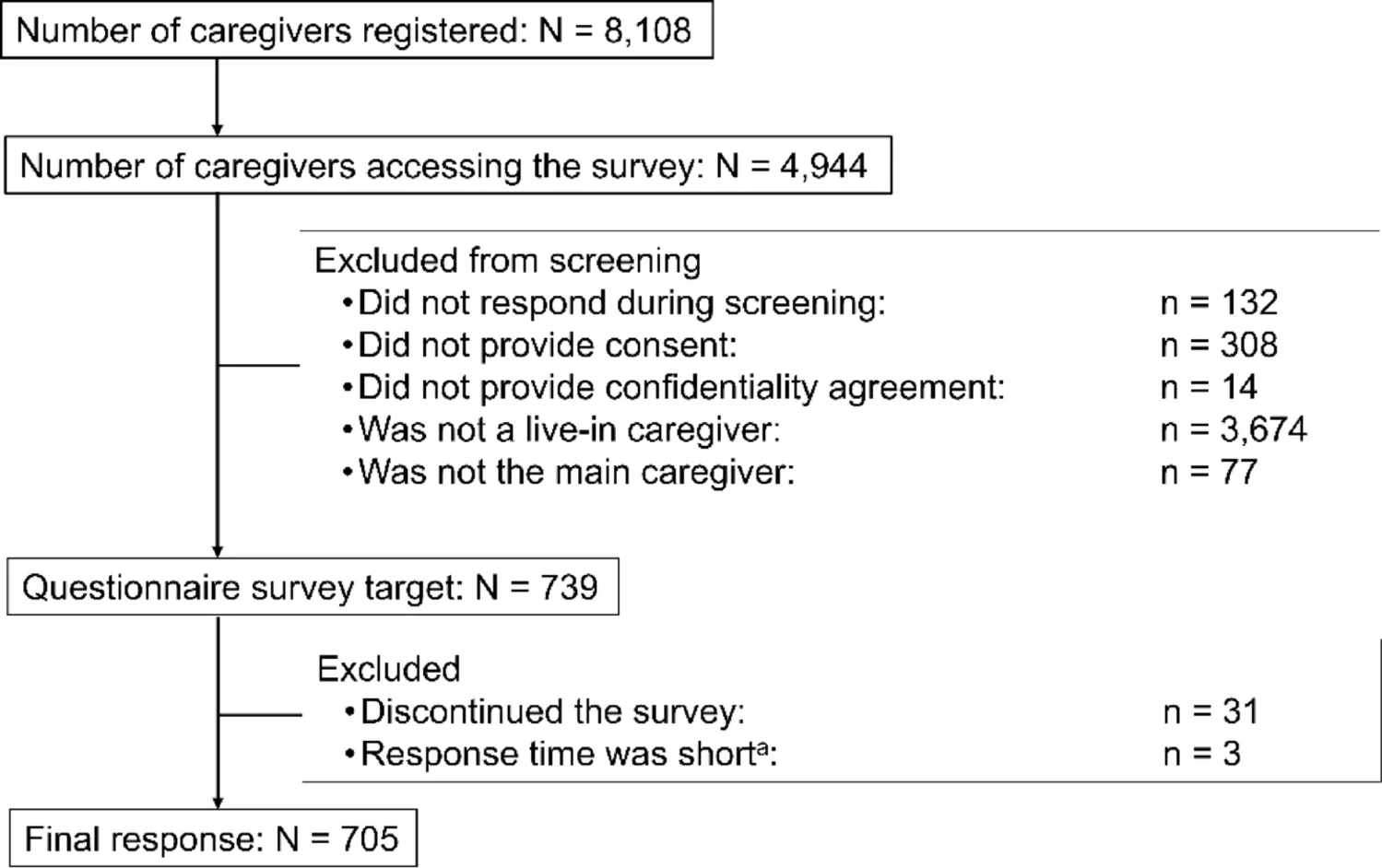
Participant flowchart from panel registration to final response. ^a^Based on the short-response-time respondent judgment criteria from Macromill, Inc

The participant flowchart is shown in Fig. 1. Of the 8108 registered caregivers, 4944 initiated the screening survey. In total, 4205 caregivers did not pass the screening process, resulting in 739 eligible caregivers for the main survey. After excluding those who discontinued the survey or had short response times, 705 live-in family caregivers of patients with AD completed the main survey.

The background characteristics of the study participants are summarized in Table 1. The mean ± SD age of the caregivers was 54.6 ± 11.5 years and 56.9% (401/705) were male. The mean ± SD J_ZBI-8, ASCOT-Carer, and EQ-5D scores were 15.5 ± 8.4, 0.71 ± 0.24, and 0.76 ± 0.19, respectively. The mean ± SD hours of care per week provided by caregivers was 24.7 ± 31.4 h. The mean ± SD age of the patients with AD was 84.2 ± 8.8 years, and 73.8% (520/705) were female.

**Table 1:** Background characteristics of patients with AD and their caregivers

Caregiver gender, *n* (%)	*N* = 705
Male	401 (56.9)
Caregiver age, years, mean ± SD Median (min, max)	54.6 ± 11.5 56.0 (20, 79)
Caregiver’s relationship to the patient, n (%)	
Spouse/partner	39 (5.5)
Siblings and siblings-in-law	3 (0.4)
Parents and in-laws	592 (84.0)
Grandparents and grandparents-in-law	65 (9.2)
Other	6 (0.9)
Primary caregiver, n (%)	
Respondent	428 (60.7)
Other family member	277 (39.3)
J_ZBI-8, mean ± SD Median (min, max)	15.5 ± 8.4 15.0 (0, 32)
ASCOT-Carer, mean ± SD Median (min, max)	0.71 ± 0.24 0.78 (0.02, 1.0)
EQ-5D-5L, mean ± SD Median (min, max)	0.76 ± 0.19 0.82 (− 0.03, 1.00)
Employment status of caregivers, n (%)	
Public servant	26 (3.7)
Business owner/executive	25 (3.5)
Company employee	238 (33.8)
Self-employed	64 (9.1)
Freelance	15 (2.1)
Full-time housewife	76 (10.8)
Part-time/temporary worker	107 (15.2)
Other	18 (2.6)
Unemployed	136 (19.3)
Caregiver’s personal income, n (%)	
Less than 500,000 yen	104 (14.8)
500,000–1 million yen	65 (9.2)
1–2 million yen	105 (14.9)
2–3 million yen	80 (11.3)
3–4 million yen	75 (10.6)
4–5 million yen	52 (7.4)
More than 5 million yen	131 (18.6)
Don’t know/Don’t want to answer	93 (13.2)
Caregiver’s highest level of education, n (%)	
Junior high school	15 (2.1)
High school	185 (26.2)
Technical college/Junior college/Vocational school	161 (22.8)
University (undergraduate)	312 (44.3)
Graduate school	29 (4.1)
Other	3 (0.4)
Hours of care per week, mean ± SD Median (min, max)	24.7 ± 31.4 14.0 (1.0, 168.0)
Patient age, years Median (min, max)	84.2 ± 8.8 86.0 (26, 101)
Patient gender, n (%)	
Male	185 (26.2)
Patient’s highest level of education, n (%)	
Common elementary school	41 (5.8)
Junior high school	196 (27.8)
High school	272 (38.6)
College of technology and junior college	79 (11.2)
Post-secondary education institution, incl. university, college, etc.	95 (13.5)
Graduate school	4 (0.6)
Other	18 (2.6)
Requiring support^a^ or long-term care^b^, n (%)	
Requiring support level 1	42 (6.0)
Requiring support level 2	31 (4.4)
Requiring long-term care level 1	162 (23.0)
Requiring long-term care level 2	167 (23.7)
Requiring long-term care level 3	122 (17.3)
Requiring long-term care level 4	75 (10.6)
Requiring long-term care level 5	63 (8.9)
Not applicable	31 (4.4)
Unknown	12 (1.7)

^a^ Patients requiring support were those able to perform most of the basic activities of daily living on their own but requiring some assistance from a family caregiver (not from a nursing care support service)

^b^ Patients requiring long-term care were those unable to perform basic activities of daily living on their own and requiring some assistance from a nursing care

Long-term care level definitions:

1. Patient’s ability to perform instrumental activities of daily living has deteriorated further from requiring support, and partial nursing care is needed

2. Significant decline in activities of daily living and instrumental activities of daily living, and partial nursing care is required for activities of daily living

3. Significant decline in activities of daily living and instrumental activities of daily living, and almost total nursing care is required

4. Patient’s ability to move is further reduced, and it becomes difficult for the patient to lead daily life without nursing care support

5. Patient’s ability to perform activities of daily living is even worse, and it is almost impossible for the patient to carry out daily living without nursing care support

### Correlation analysis and factor analysis

All 705 caregivers were included in the analysis. The absolute correlation coefficient between the J_ZBI-8 and EQ-5D-5L scores was + 0.268, that between the J_ZBI-8 and ASCOT-Carer scores was + 0.472, and that between the EQ-5D-L and ASCOT-Carer scores was + 0.463.

### Inter-factor correlation between the EQ-5D-5L and ASCOT-Carer

The anxiety and depression domain of the EQ-5D-5L showed moderate correlations with each domain of the ASCOT-Carer (r range: 0.37–0.50). The pain and the activities of daily living domains also showed moderate correlations with some domains of the ASCOT-Carer but included some domains with weak correlations. The mobility and personal care domains showed weak correlations with all domains of the ASCOT-Carer (Table 2).

**Table 2:** Correlations between the EQ-5D-5L and ASCOT-Carer

	E1	E2	E3	E4	E5	A1	A2	A3	A4	A5	A6	A7
E1	1.00											
E2	0.70	1.00										
E3	0.66	0.71	1.00									
E4	0.42	0.24	0.43	1.00								
E5	0.30	0.25	0.41	0.49	1.00							
A1	0.19	0.12	0.28	0.34	0.48	1.00						
A2	0.20	0.26	0.36	0.33	0.45	0.46	1.00					
A3	0.15	0.10	0.25	0.35	0.49	0.60	0.52	1.00				
A4	0.25	0.25	0.35	0.31	0.44	0.42	0.49	0.45	1.00			
A5	0.21	0.15	0.29	0.29	0.45	0.61	0.43	0.59	0.47	1.00		
A6	0.18	0.14	0.30	0.31	0.50	0.60	0.49	0.66	0.51	0.69	1.00	
A7	0.12	0.10	0.19	0.22	0.37	0.48	0.36	0.50	0.40	0.55	0.57	1.00

A and E stand for the dimensions belonging to the ASCOT-Carer and EQ-5D-5L, respectively

*ASCOT-Carer*, Adult Social Care Outcomes Toolkit for Carers

### Factor analysis

**Fig. 2:**
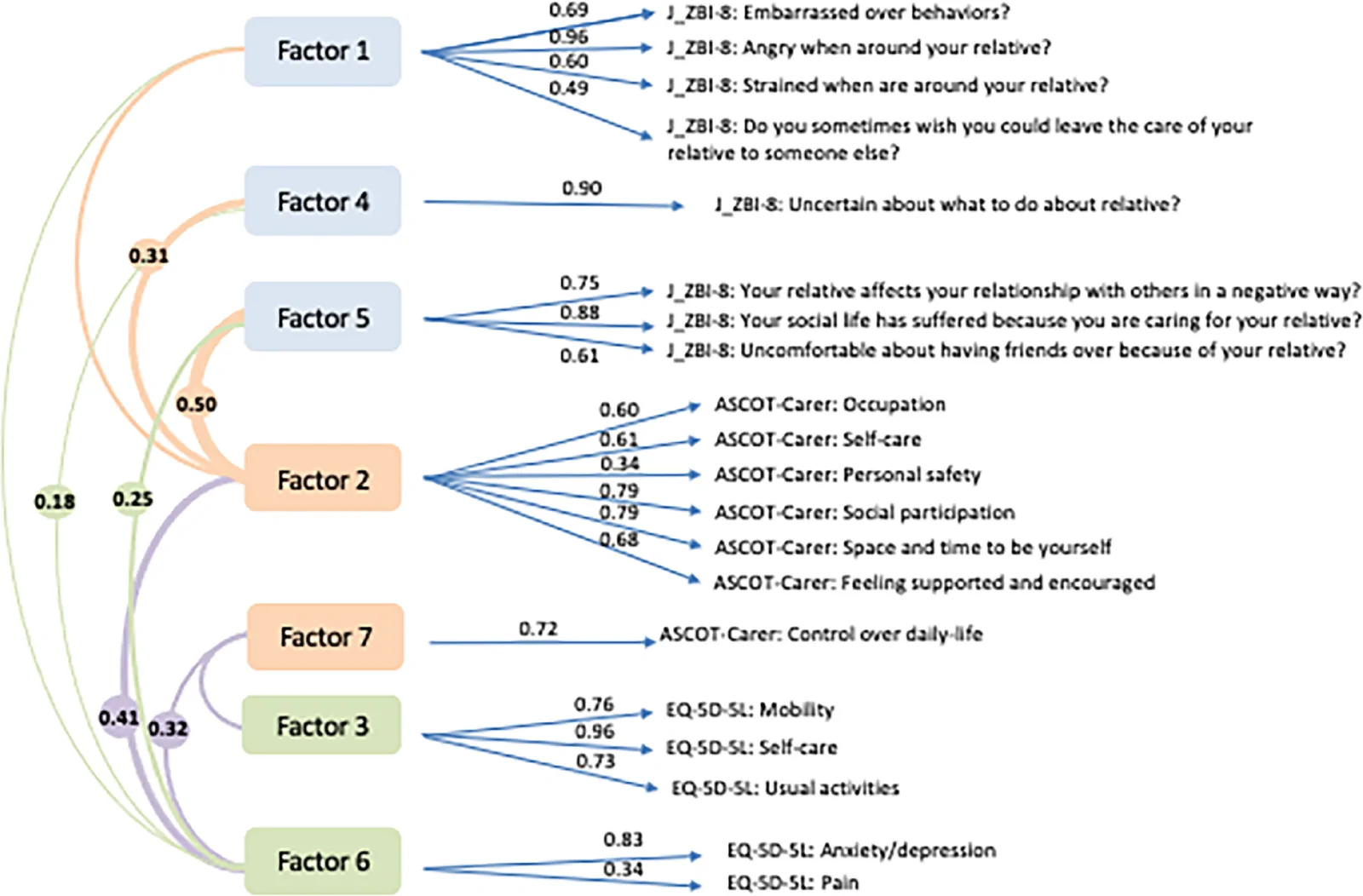
Factor loadings and correlations between the J_ZBI-8, EQ-5D-5L, and ASCOT-Carer using a seven-factor model and correlations among all the factors

The Kaiser–Meyer–Olkin measure of sample validity was 0.92, indicating that the data were appropriate for factor analysis. Figure 2; Table 3 show the results of factor loadings and correlations between the J_ZBI-8, EQ-5D-5L, and ASCOT-Carer using a seven-factor model, as well as the correlations among all the factors. Factor analysis of the 7-factor model revealed that items within each scale did not merge with items from other scales and the only factors that showed moderate or stronger correlations with the factors constituted by the J_ZBI-8 were those derived from the ASCOT-Carer (inter-factor correlation = + 0.31 and + 0.50). In contrast, factors derived from the EQ-5D-5L showed weak correlations with factors constituted by the J_ZBI-8 (inter-factor correlation = + 0.18 and + 0.25). Taken together, the factor analysis results confirm that caregiver burden aligned more closely with the ASCOT-Carer-related constructs than with EQ-5D-5L-related constructs.

**Table 3:** Factor loadings and correlations between the J_ZBI-8, EQ-5D-5L, and ASCOT-Carer using a seven-factor model and correlations among all the factors

	Factor Loadings							Communality
	Factor 1	Factor 2	Factor 3	Factor 4	Factor 5	Factor 6	Factor 7	
Z1	0.68							0.68
Z2	0.96							0.86
Z3					0.75			0.73
Z4	0.60							0.76
Z5					0.89			0.82
Z6					0.61			0.50
Z7	0.46							0.67
Z8				0.96				1.00
E1			0.75					0.69
E2			0.95					0.83
E3			0.72					0.70
E4						0.83		0.78
E5						0.34		0.53
A1		0.61						0.52
A2							0.71	0.57
A3		0.62						0.51
A4		0.35						0.45
A5		0.79						0.60
A6		0.80						0.66
A7		0.69						0.45
Correlations Among Factors								
Factor 1	1							–
Factor 2	0.29	1						–
Factor 3	0.00	0.22	1					–
Factor 4	0.76	0.31	0.12	1				–
Factor 5	0.61	0.50	0.13	0.57	1			–
Factor 6	0.16	0.41	0.39	0.18	0.25	1		–
Factor 7	0.21	0.57	0.27	0.28	0.24	0.32	1	–

Root mean square error of approximation = 0.019; Tucker–Lewis index = 0.995

*ASCOT-Carer*, Adult Social Care Outcomes Toolkit for Carers; *J_ZBI-8*, eight-item short Japanese version of the Zarit Caregiver Burden Interview

### Multivariable analysis

Point estimates and 95% CIs were calculated for both the association between caregiver burden and ASCOT-Carer and between caregiver burden and the EQ-5D-5L. The analysis revealed significant differences in gender across both indices (ASCOT-Carer: 2.06 [0.89, 3.231], *p* < 0.001; EQ-5D-5L: 2.692 [1.436, 3.948]. *p* < 0.0001), with female caregivers experiencing a significantly higher burden of care compared with male caregivers. Both indices showed a similar pattern of association with caregiver burden.

The results of the multivariable analysis of the association between caregiver burden and the ASCOT-Carer and between caregiver burden and the EQ-5D-5L are shown in *Online Resources 1*.

### Relative importance of the ASCOT-Carer and EQ-5D-5L for measuring caregiver burden

**Fig. 3:**
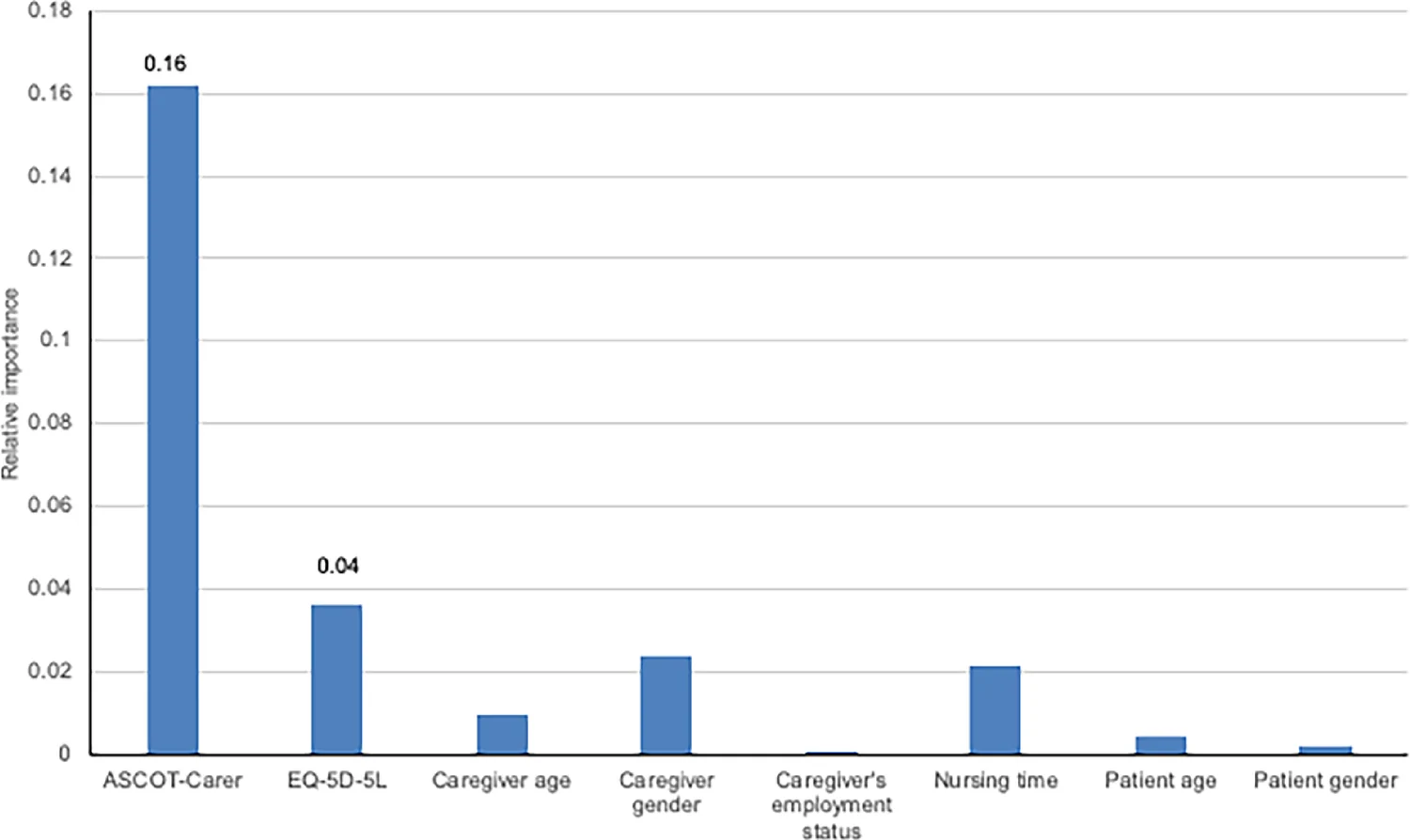
Relative importance of the ASCOT-Carer and EQ-5D-5L in measuring caregiver burden

Figure 3 shows the relative importance of the caregiver burden. The ASCOT-Carer showed a larger contribution than EQ-5D-5L for measuring caregiver’s QoL related to caregiver burden (LMG: 0.16 vs. 0.04); thus, the contribution of the ASCOT-Carer was approximately four times larger in this model. This ratio reflects R² decomposition within this specific specification and sample and does not imply causal effects or general predictive superiority.

## Discussion

In this study, data obtained from family caregivers of patients with AD in Japan were used to analyze the association between caregiver burden, as assessed using the J_ZBI-8, and QoL, assessed using the ASCOT-Carer and EQ-5D-5L. Construct overlap and empirical clustering across instruments were examined using factor analysis and inter-factor correlations (Fig. 2; Table 3), alongside total-score correlations. The results of factor analysis in this study indicated that the J_ZBI-8, ASCOT-Carer, and EQ-5D-5L scales did not converge on common factors and each had distinct underlying constructs. Notably, only the factors derived from the ASCOT-Carer showed moderate or stronger correlations with those from the J_ZBI-8, while the correlations with EQ-5D-5L factors were weak. This pattern was consistently observed across all analytical approaches employed in the Results, including correlation and regression analyses. This pattern suggests greater construct overlap between caregiver burden and SCRQoL domains than between caregiver burden and health-focused domains. These results suggest that the ASCOT-Carer is a measure of SCRQoL, while the EQ-5D-5L is a measure of health-related QoL, and that these tools capture different aspects of QoL. This finding is consistent with previous overseas studies suggesting that the EQ-5D-5L may not be suitable for evaluating the impact of care on QoL [38].

In a multivariate regression analysis with caregiver burden (J_ZBI-8) as the dependent variable and the ASCOT-Carer and EQ-5D-5L as independent variables, the ASCOT-Carer showed greater relative importance (LMG: 0.16 vs. 0.04). A higher LMG value for ASCOT-Carer, approximately four times greater than that of EQ-5D-5L, suggests that the social care-related QoL domains may capture the impact of caregiving burden on the caregiver’s QoL more appropriately than the health-focused domains measured by EQ-5D-5L. This pattern is conceptually plausible in AD caregiving, where burden is more likely to be reflected initially as restrictions in autonomy, time use, and social participation (e.g., “control over daily life,” “social participation and involvement,” and “space and time to be yourself”), with potential downstream impacts on physical and mental health when the burden is persistent and intense [51–53]. The ASCOT-Carer directly captures these aspects through domains such as managing daily life, social participation and involvement, space and time for self-realization, and sense of support, all of which are closely linked to the day-to-day impact of caregiving. In the context of caregivers of people with AD, the ripple effects of caregiving burden are particularly evident in limitations on personal autonomy, available time, and opportunities for social engagement. Consequently, the relative importance of the ASCOT-Carer in explaining caregiving burden exceeds that of the EQ-5D-5L. Taken together, these findings suggest that when the aim is to assess caregivers’ QoL through spillover effects beyond health, caregiving-related preference-based measures such as the ASCOT-Carer may provide more meaningful assessments than the EQ-5D-5L.

However, the ASCOT-Carer is not a measure of caregiver burden itself but of caregiver QoL. The results of this study suggest that the ASCOT-Carer may be a useful tool for understanding the impact of caregiver burden on caregiver QoL, and appropriate scale selection based on research objectives is required for its application.

Health economic evaluations have mainly focused on the affected individuals, ignoring spillover effects [54]. However, these have evolved in recent years, and studies that have evaluated spillover effects on caregivers and others have used common PBMs such as the EQ-5D, Short-Form 6-Dimension (SF-6D), and Health Utilities Index (HUI) [17]. With the development of the ASCOT-Carer, a care-related PBM, future use of this assessment tool is expected to increase [55].

The present study findings complement existing research, indicating that the ASCOT may be more sensitive than the EQ-5D in studies where the purpose of the intervention is broader than simply improving or maintaining health [56]. This suggests that the ASCOT-Carer may be more sensitive than the EQ-5D-5L in capturing the impact of caregiving burden on the QoL of caregivers of patients with AD. Such differences are consistent with the Carer Stress and Burden Model [57], which explains the relationship between caregiving stress and QoL. According to this model, caregiver burden initially affects their subjective well-being and QoL. When this stress persists over a prolonged period and at high intensity, it can ultimately have a negative impact on their physical and mental health. This model provides a plausible explanation for why caregiving-related impacts may be reflected more strongly in care-related QoL domains than in health-focused domains.

Other PBMs for caregivers include the CarerQoL and the CES instrument [30, 31]. Prior research has shown that existing scales capture different constructs, even among PBMs that aim to measure the same caregiver’s QoL [58]. The CES and CarerQoL seem better suited to investigate changes directly related to caregiving status, while the ASCOT-Carer measures a broader range of QoL domains outside of caregiving [58]. PBM selection should be guided by the evaluation objective and the outcome domain of interest.

### Strengths and limitations

Regarding the study strengths, this is the first study to examine the appropriateness of the ASCOT-Carer in measuring the impact of caregiver burden on the QoL among informal caregivers of patients with AD in Japan. The study findings on the appropriateness of the ASCOT-Carer vs. EQ-5D-5L in measuring the burden of care may be valuable in developing future guidance for using scales in health economic evaluations.

This study has some limitations. Caregivers of patients with AD in Japan who were family members and were living together with the patients were targeted, which limits the generalizability of the findings to caregivers of patients with other conditions, from different regions, or with other characteristics (e.g., those unrelated to the patient). The target population was limited to live-in family caregivers, whose caregiving circumstances tend to be relatively uniform. The impact of caregiving experience on QoL may differ for live-out caregivers, who may encounter caregiving demands that differ with respect to care coordination, time commitment, and role obligations. Future research should examine whether the observed associations between caregiver burden and QoL, as well as the measurement characteristics of the ASCOT-Carer and EQ-5D-5L, are consistent in live-out caregiving settings. Furthermore, because participants were drawn from an opt-in online panel, self-selection bias cannot be excluded (e.g., individuals more willing to participate in online surveys may differ systematically from those who do not participate).The incentive of completing the survey may have encouraged unwarranted responses. Selection bias is a concern because it was a web-based survey. In particular, older caregivers may have been less likely to participate in or complete an online survey, which could have resulted in under-representation of older caregivers in our sample. All data were collected via self-reporting and may have been subject to social desirability bias; caregivers may have under-reported their burden or over-reported their QoL. Therefore, there is a risk that the results may have underestimated the true impact. In addition, detailed information on the use, type, and intensity of formal care services was not collected, and these unmeasured factors may influence both caregiver burden and quality of life. Therefore, the generalizability of the findings to the broader Japanese caregiver population should be interpreted with caution.

## Conclusions

The ASCOT-Carer may be a more suitable tool for evaluating the impact of caregiving burden on caregivers’ QoL. This suggests that when incorporating QoL based on caregiver burden in health economic evaluations, the ASCOT-Carer, which is a PBM, may be an appropriate option. 

## Supplementary Information

Below is the link to the electronic supplementary material.

Supplementary Material 1

## Data Availability

The participants in this study did not provide written consent for their data to be shared publicly; therefore, the supporting research data are not available.

## Abbreviations

AD: Alzheimer’s disease
ASCOT-Carer: Adult Social Care Outcomes Toolkit for Caregivers
J_ZBI-8: Eight-item short Japanese version of the Zarit Caregiver Burden Interview
SD: Standard deviation
