# Supplementary material for: Potential of ASCOT-Carer for evaluating the quality of life of family caregivers for patients with Alzheimer’s disease in Japan
Source: Qual Life Res. 2026 May 3;35(6):141. doi: 10.1007/s11136-026-04252-6 (PMC13136206; doi:10.1007/s11136-026-04252-6)
Supplement: Supplementary file 1 — Supplementary Material 1 [file 11136_2026_4252_MOESM1_ESM.docx]

**Supplementary Materials**

**Online Resource 1.** Multivariable analysis of the association between caregiver burden and the ASCOT-Carer and between caregiver burden and the EQ-5D-5L

|  | Between  caregiver burden and the ASCOT-Carer | Between  caregiver burden and the EQ-5D-5L |
| --- | --- | --- |
| N | 697 | 697 |
| Akaike’s Information Criterion | 4806 | 4898 |
| Bayesian Information Criterion | 4847 | 4939 |
| ASCOT-Carer |  |  |
| Estimate (95% CI) | −14.988 (−17.358, −12.617) | −10.049 (−12.854, −7.245) |
| p-value | <0.0001 | <0.0001 |
| Age of patient with AD |  |  |
| Estimate (95% CI) | −0.045 (−0.112, 0.023) | −0.048 (−0.121, 0.024) |
| p-value | 0.198 | 0.192 |
| Gender of patient with AD (female: control = male) |  |  |
| Estimate (95% CI) | −0.121 (−1.368, 1.126) | −0.861 (−2.184, 0.463) |
| p-value | 0.849 | 0.202 |
| Age of caregiver |  |  |
| Estimate (95% CI) | −0.046 (−0.1, 0.009) | −0.049 (−0.108, 0.01) |
| p-value | 0.103 | 0.101 |
| Gender of caregiver (female: control = male) |  |  |
| Estimate (95% CI) | 2.06 (0.89, 3.231) | 2.692 (1.436, 3.948) |
| p-value | 0.001 | <0.0001 |
| Caregiver’s employment status (Yes: control = no) |  |  |
| Estimate (95% CI) | −0.571 (−1.79, 0.647) | 0.493 (−0.809, 1.794) |
| p-value | 0.358 | 0.457 |
| Nursing time |  |  |
| Estimate (95% CI) | 0.014 (−0.005, 0.033) | 0.045 (0.026, 0.064) |
| p-value | 0.141 | <0.0001 |

Abbreviations: *AD*, Alzheimer’s disease; *ASCOT-Carer*, Adult Social Care Outcomes Toolkit for Carers; *CI*, confidence interval.
